# Optimizing Vaccination Strategies against African Swine Fever Using Spatial Data from Wild Boars in Lithuania

**DOI:** 10.3390/v16010153

**Published:** 2024-01-19

**Authors:** Vincenzo Gervasi, Marius Masiulis, Paulius Bušauskas, Silvia Bellini, Vittorio Guberti

**Affiliations:** 1 Istituto Superiore per la Protezione e la Ricerca Ambientale, Via V. Brancati 60, 00144 Roma, Italy; 2 State Food and Veterinary Service of the Republic of Lithuania, Siesiku 19, 07170 Vilnius, Lithuania; marius.masiulis@vmvt.lt (M.M.); paulius.busauskas@vmvt.lt (P.B.); 3 Veterinary Academy, Lithuanian University of Health Sciences, Tilzes 18, 47181 Kaunas, Lithuania; 4 Istituto Zooprofilattico della Lombardia ed Emilia-Romagna, Via A. Bianchi 7/9, 25124 Brescia, Italy; silvia.bellini@izsler.it; 5 Istituto Superiore per la Protezione e la Ricerca Ambientale, Via Cà Fornacetta 9, 40064 Ozzano dell’Emilia, Italy; vittorio.guberti@isprambiente.it

**Keywords:** *Asfaviridae*, disease eradication, home range size, oral mass vaccination, *Sus scrofa*

## Abstract

African swine fever (ASF) is one of the most severe suid diseases, impacting the pig industry and wild suid populations. Once an ASF vaccine is available, identifying a sufficient density of vaccination fields will be crucial to achieve eradication success. In 2020–2023, we live-trapped and monitored 27 wild boars in different areas of Lithuania, in which the wild boars were fed at artificial stations. We built a simulation study to estimate the probability of a successful ASF vaccination as a function of different eco-epidemiological factors. The average 32-day home range size across all individuals was 16.2 km^2^ (SD = 16.9). The wild boars made frequent visits of short durations to the feeding sites rather than long visits interposed by long periods of absence. A feeding site density of 0.5/km^2^ corresponded to an expected vaccination rate of only 20%. The vaccination probability increased to about 75% when the feeding site density was 1.0/km^2^. Our results suggest that at least one vaccination field/km^2^ should be used when planning an ASF vaccination campaign to ensure that everyone in the population has at least 5–10 vaccination sites available inside the home range. Similar studies should be conducted in the other ecological contexts in which ASF is present today or will be present in the future, with the objective being to estimate a context-specific relationship between wild boar movement patterns and an optimal vaccination strategy.

## 1. Introduction

The spread of African swine fever (ASF) to several European, Asian, and more recently, central American countries [1,2,3,4] represents a serious threat to the economic system related to pork meat production worldwide. ASF is also a strong demographic pressure on all wild pig populations in these continents [5,6]. ASF is due to a highly virulent and structurally complex virus of the *Asfaviridae* family (ASFV genotype II), which affects wild boars (*Sus scrofa*), domestic pigs and other wild suid species [7,8], leading to almost 100% lethality in the infected individuals [8] and to a sharp reduction in population densities.

During the last 15 years of ASF invasion into more than 35 previously unaffected countries, experience has shown that eradication is possible, especially during the early phases of the invasion process. Eradication chances are higher if quick and effective confinement measures, such as fencing and zone-based restrictions [9], are quickly put in place well before the affected area becomes too large to be managed [10,11]. ASF is otherwise very likely to become endemic in wild boar populations [12] and the chances of eradicating the disease at a later stage through depopulation are very low [13].

Due to the structural complexity of the ASFV, the efforts produced by the international scientific community to produce a safe and effective vaccine for this disease have not been successful so far. Progress has been made, though, toward the identification of suitable vaccine candidates. Teklue et al. [14] reported that six different experimental live attenuated vaccines have been tested by different research groups in the period 2015–2018, all with various performance and safety issues. More recently, further advancements have been published by Barasona et al. [15] and Chen et al. [16], who reported almost 90% protection against challenge with a virulent ASFV isolate in domestic pigs, although it was associated with some risk of disease development in case of overdosing [17]. Barasona et al. [15] also presented an experimental study performing oral immunization in wild boar with a non-hemadsorbing, attenuated ASF virus of genotype II isolated in Latvia in 2017 (Lv17/WB/Rie1). The vaccine conferred 92% protection against challenge. Deutschmann et al. [18], instead, presented a different vaccine candidate, resulting in 50% seroconversion after oral immunization in wild boar and full protection after intramuscular injection in pigs. The authors of both works called for future studies to assess the vaccine’s safety following repeated administration or overdose, its genetic stability during passages, its stability in the field, and its differentiability from the infecting virus based on DIVA serological testing [15,18].

Once an effective and safe vaccine is available, at least 40% (but preferably >50%) of the wild boar population should be vaccinated for at least 1–2 years to provide a high probability of successful disease eradication. In the case of classical swine fever (CSF), the use of palatable baits for oral mass vaccination at pre-baited feeding sites was considered the most satisfactory option. This methodology allowed researchers to improve the likelihood of reaching the desired vaccination rates over extensive geographical areas [19]. It is likely that the same type of strategy will be employed also for a foreseeable ASF vaccination campaign.

One of the crucial elements of achieving the necessary ASF vaccination coverage will be the way the vaccination campaign will be designed in terms of the total effort, spatial arrangement of the vaccination fields, frequency and duration of the bait distribution events, etc. Previous experiences with CSF have shown that identifying the appropriate timing of the vaccination and a sufficient density of baited fields is strongly related to eradication success [20]. These factors, though, are not expected to be constant among the different geographical contexts and populations. They are expected to depend on several ecological parameters, such as the wild boar density, landscape structure, and most of all, on the patterns of animal space use during the year [21]. Therefore, being able to fine tune the characteristics of a vaccination campaign on the specific movement patterns of the target population would be a crucial advantage for improving the effectiveness and efficiency of vaccination.

**Figure 1:**
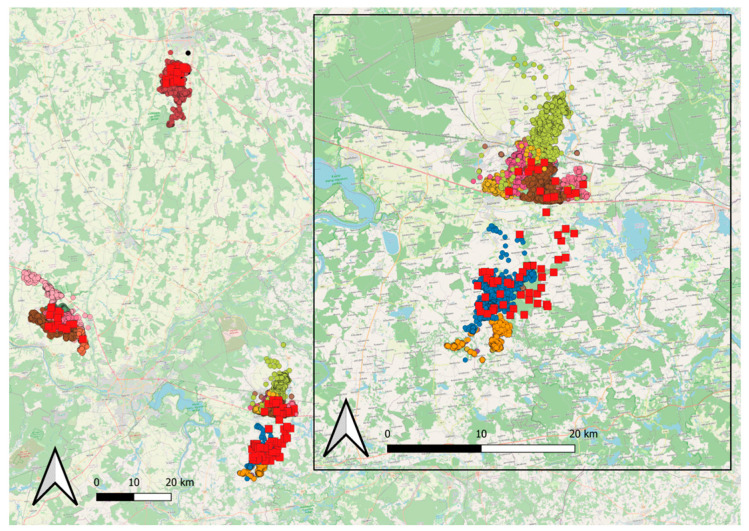
Spatial distribution of all the GPS locations (circles of different colors refer to different individuals) collected for 27 wild boars captured in Lithuania in the period 2020–2022 and monitored through GPS–GSM collars. The red squares represent the spatial distribution of the wild boar artificial feeding sites. The inset shows the distribution of the GPS locations in one of the three areas.

During the period 2020–2023, the State Food and Veterinary Service of the Republic of Lithuania (SFVS) conducted a field study based on live-trapping and Global Positioning System (GPS) collar-monitoring of wild boars in different ASF-affected areas in Lithuania (Figure 1).

The project produced a substantial amount of information about wild boar space use patterns in one of the geographic areas first affected by ASF after its first introduction into Europe in 2007 [3,22] and in which the disease has become nowadays endemic. This represented a suitable context and a valuable dataset to explore the issues related to the design of a future ASF vaccination campaign for wild boar populations.

In this paper, we present the results of a combined analysis, based on both field-based data and simulated data, to assess the expected performance of an ASF vaccination campaign in Lithuania. We first estimated the main parameters describing wild boar space use in Lithuania using GPS-based spatial data; then, we built a simulation study to estimate the probability of a successful ASF vaccination in wild boars as a function of different ecological and design-related factors. We discuss the results of our study in the general context of how vaccination could be used as an effective ASF eradication tool in Europe, providing indications of how such an effort such be appropriately planned and realized.

## 2. Materials and Methods

### 2.1. Live Trapping and GPS Monitoring of Wild Boar

We used three types of live traps for wild boar capture: metal cage traps, wooden box traps, and wire net traps. The size of the metal and wooden box taps was approximately two meters in width, one meter in height and one meter in depth, and they were designed for a small number of wild boars to be trapped. The metal wire traps differed in size, depending on the limitations and possibilities related to the specific trapping site, but their size ranged approximately from three to four meters in length, two to three meters in depth, and 1.5 to two meters in height. The traps were equipped with 90–100 cm wide guillotine-style single-catch wooden doors. The doors were triggered by wild boars activating a trapwire. We monitored all the trapping sites using camera traps (Reolink Go Plus, Reolink, Shenzhen, China). We selected the trapping sites based on the presence of wild boars in the hunting ground, where they were usually baited with maize two to three weeks before the actual trapping effort took place. We obtained all the permissions to trap and release collared wild boars from the Lithuanian Ministry of Environment and from hunting ground managers.

The trapping team consisted of three veterinarians, who performed the animal handling, sedation, and who collared the wild boars following animal welfare regulations. The average time between the trap triggering and the arrival of the trapping team was 1–2 h, whereas the entire procedure from anesthesia to releasing the collared wild boar lasted a maximum of 15–20 min. For anesthesia, we used Zoletil^®^100 (Virbac, Carros, France; 250 mg tiletamine and 250 mg zolazepam) with a targeted doze of 3.75 mg/kg, using The DANiNJECT Jab Stick (DANiNJECT, Kolding, Denmark) for the intramuscular injection of the drug into the hindquarters. We calibrated the anesthetic dose after visually estimating the body weight of the captured animals.

We monitored the long-term welfare of the collared animals using game camera traps located at the baiting sites and along regularly used wild boar trails in the study area, as well as through the OrniTrack movement analysis program developed for wild boar monitoring purposes, which includes the animal temperature and movement frequency (OrniTrack Control Panel, Ornitela, Vilnius, Lithuania). We did not test the captured wild boars for ASFV because, given the very high mortality of this disease, infected animals were expected to die a few days after incubation and to be retrieved using the mortality signal included in the GPS collar. Accordingly, all the hunted wild boars and those found dead were tested for ASFV following the standard surveillance protocols.

We used two types of Global Positioning System (GPS) collars produced for wild boar movement tracking (Ornitela UAB, Vilnius, Lithuania). The GPS collars had no drop-off function. All the collars had an internal antenna for signal transmission via GSM and GPRS and an expected battery duration of at least 12 months. Therefore, all the data were transmitted from the collar via the GSM network. We recorded the animals’ GPS position at least two times per hour, recording at least 24 GPS positions per day.

### 2.2. Data Analysis

After compiling all the GPS data, we removed from the final dataset all the individuals monitored for less than 14 days, because we considered the dataset too small to produce reliable information. Then, we produced a series of descriptive statistics to start exploring the patterns of space use by wild boar in the study area. In particular, we focused on the use of the artificial feeding stations, which would represent the optimal sites for vaccine bait distribution. To achieve this aim, we first estimated the individual home range size during all the 32-day GPS location sequences available for each wild boar (see below for the choice of 32 days as the period for the home range estimation). We estimated the individual home range as the minimum convex polygon (MCP) of all the GPS locations referring to each 32-day monitoring period. Then, for each individual home range, we calculated the number of feeding stations included in the area used by the wild boar and potentially suitable for vaccination. Finally, we calculated the linear distance between each GPS location and all the feeding stations in the home range to assess how often a wild boar approached a feeding area and how long it remained in its proximity. We considered that a wild boar visited a given feeding station when it was detected less than 100 m from the site. The distance threshold accounted for the need to consider the potential inaccuracy of the GPS locations, especially in forest habitats. Sager-Fradkin et al. [23] reported an average location error of 62.6 m for GPS collars in the temperate coniferous forests of Washington state, USA. For these and all the other analyses, we merged juvenile (<12 months) and adult (>24 months) individuals into a unique group, as we considered the GPS locations derived from a juvenile wild boar to be representative of its mother’s space use patterns. Yearling individuals (12–14 months of age) were treated separately.

After performing the initial data exploration, we set up a series of simulated vaccination scenarios. The simulations were based on the distribution of the actual wild boar GPS locations and feeding stations and on a vaccination schedule that resembled the one adopted for classical swine fever during the late 1990s of the last century and early years of the 2000s [20]. In that case, the vaccination process was based on three double campaigns in spring, summer, and autumn. Each campaign comprised two vaccine–bait distributions spaced by 28 days. Experimental studies had shown that a first vaccination followed by a booster after 28 days is the solution maximizing immunization [24]. 

To mimic the same type of vaccination campaign, we simulated the distribution of vaccine baits at all the feeding sites included in the home range of each wild boar for each day it was monitored through a GPS collar. For instance, we started simulating the bait distribution on day 1 at all the feeding sites included in the home range of the first wild boar. Then, a site was considered as visited (and the vaccine bait ingested) if the animal approached closer than 100 m from the site within the following five days. This choice was based on the evidence, derived from the CSF vaccination campaign, that wild boars developed protection against challenge about 4 days after application of the vaccine and that the oral bait is likely to persist in the forest environment only for a few days after its distribution [25]. We repeated the simulated bait distribution on day 28 and allowed all the sites to be visited by the same animal until day 32. Only if the wild boar visited at least one baited site in both sessions was it considered as fully vaccinated. Then, we repeated the same procedure for all the days an individual was GPS-monitored, thus simulating the outcome if vaccination started on day 2, 3, 4 and so on, and for all the individuals in the sample. 

The complete simulation exercise produced a dataset with the identity of each wild boar, its sex and age class, the density of the baiting stations in its home range (n. stations/km^2^), the first day of the vaccine distribution, and the outcome of the vaccination process (1 = animal fully vaccinated; 0 = animal not vaccinated). We used this dataset to build and analyze a mixed-effects logistic regression model with the individual wild boar as a random effect to account for pseudo-replication [26]. We used the vaccination outcome as a response variable and estimated the vaccination probability as a function of the sex, age, density of feeding stations, and day of the year. As we expected the vaccination probability to have an optimum during a certain season of the year, we included both a linear and a quadratic effect of the time variable to allow for such type of mathematical relationship. We performed all the analyses in R 4.2.1.

## 3. Results

Between November 2020 and April 2022, we captured and monitored 32 wild boars. Out of them, 5 were monitored for less than 14 days (3 because of a malfunctioning of the GPS collar and 2 because they were hunted a few days after being radio-collared). These individuals were removed from the dataset. Out of the remaining 27 individuals, 22 were males and 5 were females, 4 were juveniles, 12 yearlings and 11 were adults (see Table S1 in the Supporting Information for details). The average number of GPS monitoring days per individual was 120.2 (SD = 82.0, min = 22, max = 292). The average number of GPS locations per individual was 3224 (SD = 2626, min = 423, max = 4640).

**Figure 2:**
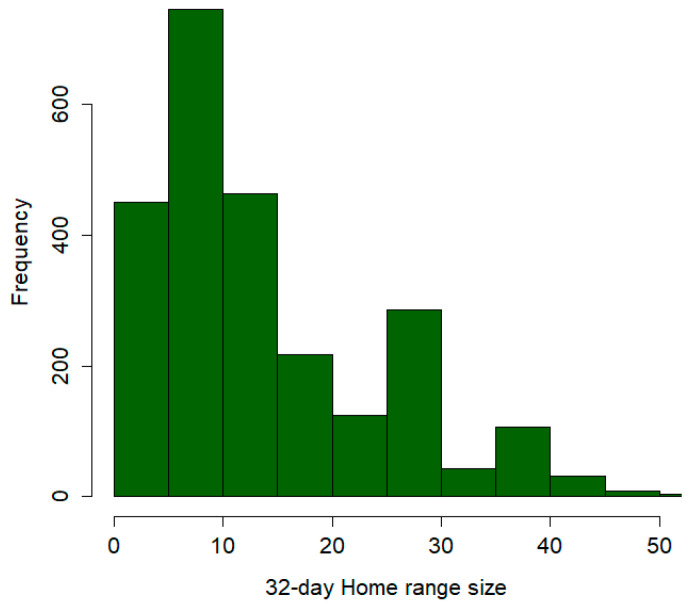
Frequency distribution of the home range size estimates for 27 wild boars captured in Lithuania in the period 2020–2022 and monitored through GPS–GSM collars. The estimates are based on groups of GPS locations referring to a 32-day period. The *x*-axis reports the size of individuals’ home ranges expressed in km^2^.

Using the 87,058 total GPS locations and the 3246 GPS monitoring days, we defined 2576 32-day monitoring windows, which we used for the home range size estimation and for all the subsequent simulations. The average home range size across all the individuals and time windows was 16.2 km^2^ (SD = 16.9, min = 0.4, max = 113.8; Table 1 and Figure 2). 

Adult males exhibited the largest average home range size among all the groups (mean = 17.4, SD = 19.1), whereas adult females were the group with the smallest size (mean = 9.1, SD = 4.7), although their estimate was based only on two individuals.

**Figure 3:**
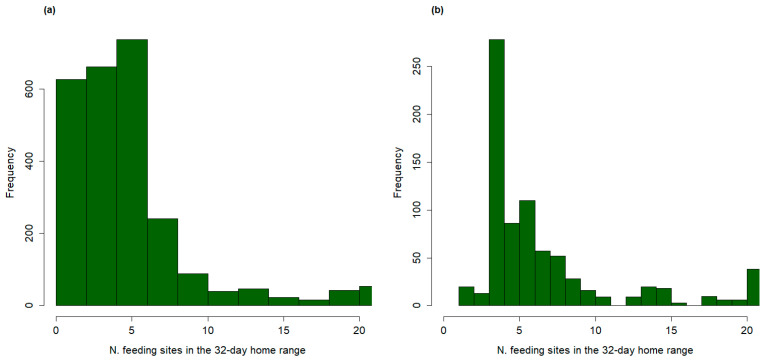
Frequency distributions of the number of artificial feeding sites contained in the 32-day home ranges (reported on the *x*-axis) of 27 wild boars captured in Lithuania in the period 2020–2022 and monitored through GPS–GSM collars. The frequency distribution is shown for all the home ranges (**a**) and for the subset of home ranges associated to a successful simulated vaccination against ASF (**b**).

Out of the 2576 estimated home ranges, 352 contained no feeding points and had, therefore, no vaccination probability. The average number of feeding sites per home range was 5.13 (SD = 4.44), with a large variation among individuals and periods of the year. In fact, 24% of the estimated home ranges included less than four feeding sites, whereas 15% contained more than eight feeding sites (Figure 3a). The resulting average density of feeding sites among all the estimated 32-day home ranges was 0.32 sites/km^2^.

By breaking the wild boar trajectories into segments between successive visits to any feeding site, we identified 302 intervals. Out of them, in 206 cases (68% of the total), the wild boar remained in proximity to the site for only one day and then moved away (Table 2); in 31 cases (10%), the animal stayed at the site for two days; in 30 cases (10%), for 3–4 days; whereas in 35 cases (12%), it stayed around the site for more than four days.

When considering the time interval between successive visits to feeding sites, the analysis revealed that in 105 cases, corresponding to 35% of the total, the animal stayed away from any feeding site for two days before visiting a new one (Table 2); in 55 cases (18%), the interval was three days; in 25 (8%), four days; and in the remaining 117 cases, (39%) more than four days. Overall, this part of the analysis revealed that the most common pattern of use of feeding sites was that of very frequent visits of short durations rather than long visits interposed by long periods of absence. When considering the whole dataset, wild boars visited a feeding site in 769 days out of the 3246 monitoring days, corresponding to 23.7% of the total.

Out of the 2576 simulated iterations, 786 (30.5%) resulted in a successful wild boar vaccination, meaning that the individual visited a feeding site during both 5-day intervals separated by a 28-day gap. In 862 iterations (33.5%), the result was a partial vaccination, corresponding to only one visit to a feeding site during the vaccination period. The remaining 928 iterations (36.0%) resulted in a vaccination failure. The logistic regression model revealed a significant effect of all the variables included as predictors (Table 3).

**Figure 4:**
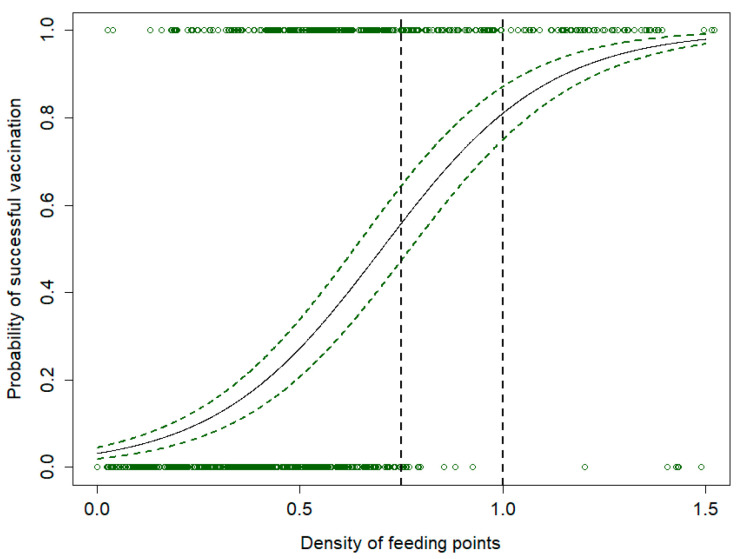
Relationship between the number of artificial feeding sites per km^2^ within a 32-day wild boar home range and the probability of successful vaccination of wild boars in Lithuania, resulting from a simulation model and a binomial generalized linear mixed model.

As expected, the density of feeding sites inside an individual home range was a strong predictor of vaccination success. Among the groups tested, adult females were the one with the lowest vaccination probability, whereas male yearlings the one with the highest probability (Table 3). As shown in Figure 4, a feeding site density of 0.5/km^2^ corresponded to an expected vaccination rate of only 20%. The vaccination probability increased to about 50% for a density of 0.75 sites/km^2^ and to about 75% when the feeding site density was 1.0/km^2^.

**Figure 5:**
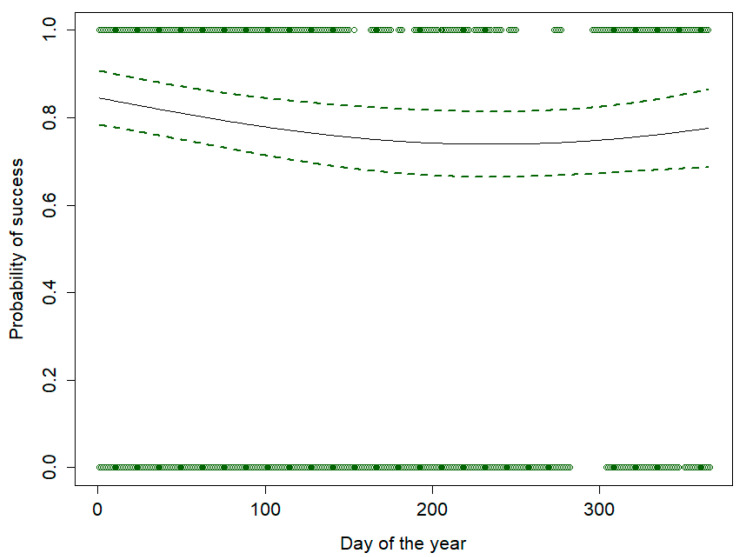
Relationship between the day of the year and the probability of successful vaccination of wild boars in Lithuania, resulting from a simulation model and a binomial generalized linear mixed model.

The model also revealed a significant but moderate effect of the period of the year on the vaccination probability. Keeping constant all the other factors, the vaccination rate was slightly higher at the beginning and at the end of the year, i.e., from late autumn to early spring, whereas it was moderately lower in summer (Figure 5). Although statistically significant, the effect size is not expected to play a major role in practical terms. 

Considering only the iterations resulting in a full vaccination, the average number of feeding sites contained in a wild boar home range was 7.2 (SD = 4.9), higher than the 5.1 calculated for all the iterations. Moreover, in 28.5% of the successful vaccinations, the individual home range contained more than 8 feeding sites, in 67.5% of the cases the wild boar home range contained 4–7 feeding sites, and only in 4% of the cases less than 4 sites (Figure 3b).

This reveals that having at least 4 feeding sites inside the area used during a 32-day period was crucial to provide a non-negligible probability of being vaccinated. Overall, the average density of feeding sites inside the home ranges that resulted in a successful vaccination was 0.68/km^2^ (SD = 0.29), more than double than the average of 0.32 sites/km^2^ estimated for both the successful and unsuccessful iterations.

## 4. Discussion

Previous experience with the classical swine fever vaccination campaigns has proven that controlling this type of wildlife disease over extensive geographical areas through vaccination is possible when an effective vaccine is available, but only through a great collective effort and a carefully defined vaccination design repeated for several years in a row [20]. At present, the main limitation of such direction for ASF control is the lack of a vaccine with high standards of efficacy and security [14]. Still, understanding the interactions between the medical and the ecological component of the vaccination process is a crucial requirement. With this aim, our study tried to make the most out of a large dataset of wild boar GPS locations in an ASF-affected area and provide a set of quantitative information about the patterns of wild boar space use in Lithuania, which can substantially contribute to informing a future vaccination program once the vaccine is available. The relatively large number of wild boars monitored allowed us to capture wide differences in the individual home range size during the study period, as the smallest home range was 0.4 km^2^, whereas the largest was 113 km^2^. Therefore, the simulated vaccination designs were tested on a broad spectrum of different individual movement patterns in three areas with similar habitat characteristics, corresponding to a boreal forest interspersed with clearcuts and human infrastructures.

In general, wild boars in Lithuania were shown to use relatively large areas in short time periods, considering that the average home range size over a period of about a month was larger than 15 km^2^. This is in line with the relatively low productivity of forest habitats in boreal areas. In a more productive, mediterranean environment, Massei et al. [27] estimated an average monthly home range size ranging from 1.2 to 1.8 km^2^ for wild boars in Tuscany, central Italy. Similarly, Fattebert et al. [28] reported a seasonal home range size estimate of about 4.0 km^2^ in Switzerland. Despite the supplemental feeding, wild boars in Lithuania exhibited the need to move over much larger areas to satisfy their needs. Accordingly, the estimated home range size values are more in line with the 33 km^2^ reported for wild boars at the northern edge of their distribution, close to the border between Finland and Russia [29]. Large monthly movements can represent a positive factor for vaccination, because animals that move a lot are more likely to visit at least one vaccination site during bait distribution, provided that the network of vaccination sites is large and dense enough in relation to animal movement patterns. The main issue when vaccinating wildlife is represented by animals with small home ranges and short movements in areas with low density of vaccination sites, because such animals are exposed to a low number of vaccination sites and have a higher risk of visiting none of them during the short time window in which vaccine baits are available and effective. Accordingly, adult females were found to be the age and sex class with the smallest home range size, and the group with the lowest vaccination probability.

In terms of the use of feeding sites, wild boars in Lithuania were shown to rely consistently on artificial feeding, as they spend one-fourth of their time in proximity to a feeding station. In general, they visit these sites often, stay for short periods (1–2 days) and then move away, likely visiting the same or another feeding sites after a few days. This is also a positive pattern of space use when evaluated through the lens of a possible future vaccination campaign, because frequent short visits to vaccination sites contribute to increasing the vaccination probability, especially if two bait ingestions will be necessary to complete the vaccination and produce immunization.

Overall, the simulation study showed that the density of feeding sites currently available in the study area (0.32 sites/km^2^) is likely to be not sufficient to ensure a satisfactory vaccination rate in the case of ASF vaccination. Accordingly, only 30% of the simulated vaccination trials resulted in a successful vaccination, while a threshold of 40–50% is considered the minimum proportion of the population to be vaccinated to ensure a realistic chance of disease eradication [30]. Our simulation results, in line with what was previously performed for CSF, suggest that at least one vaccination field/km^2^ should be used when planning and performing a foreseeable ASF vaccination campaign to ensure that everyone in the population has at least 5–10 vaccination sites available inside the monthly home range. Lower densities of vaccination fields could produce low vaccination rates, thus reducing the epidemiological effects of a massive logistic and economic effort.

As mentioned above, the relationship between wild boar space use and vaccination success is expected to be highly context dependent, as witnessed by the large variation in the home range sizes reported above. Therefore, while our study provides general indications of how to design an ASF vaccination campaign, and some specific indications of how to implement it in the boreal ecosystems of north-eastern European countries, we suggest that similar studies be conducted in the other ecological contexts in which ASF is present today or will be present in the future, with the objective being to estimate a context-specific relationship between wild boar movement patterns and an optimal vaccination strategy. Our study presents a methodology that can be applied in any situation in which wild boar movement data are available.

## Figures and Tables

**Table 1:** Sex- and age-specific home range size estimates for 27 wild boars captured in Lithuania in the period 2020–2022 and monitored through GPS–GSM collars. The estimates are based on groups of GPS locations referring to a 32-day period.

Group	N. Wild Boars	Home Range Size (km^2^)	
		Average	SD
Adult females	2	9.1	4.7
Adult males	13	17.4	19.1
Yearling females	3	10.4	7.4
Yearling males	9	10.4	7.4
Total	27	16.2	16.9

**Table 2:** Summary of the number of days between successive wild boar visits to a feeding site and the time spent in proximity (<100 m) to a site. The figures refer to the number of GPS sequences falling in the correspondent category.

N. Days	Time between Successive Visits	Time Spent at the Site
1	-	206
2	105	31
3	55	14
4	25	16
5	12	7
6	17	4
7	5	4
8	10	4
9	9	5
10	4	3
>10	60	7

**Table 3:** Parameter estimates of a binomial generalized linear mixed model estimating the probability of full vaccination as a function of the wild boars’ individual characteristics, density of feeding sites and period of the year.

Parameter	Estimate	SE	*p*-Value
Intercept	−3.163	0.265	<0.001
Feeding sites density	4.866	0.243	<0.001
Sex (female)	0.387	0.146	0.008
Age class (adult)	0.452	0.117	<0.001
Day	−0.056	0.002	0.007
Day^2^	0.001	0.0005	0.035

## Data Availability

The raw data supporting the conclusions of this article will be made available by the authors on request.

## Supplementary Materials

The following supporting information can be downloaded at https://www.mdpi.com/article/10.3390/v16010153/s1, Table S1: Individual characteristics and monitoring patterns of 27 wild boars captured in Lithuania in the period 2020–2022 and monitored through GPS–GSM collars.
