# Supplementary material for: Optimizing Vaccination Strategies against African Swine Fever Using Spatial Data from Wild Boars in Lithuania
Source: Viruses. 2024 Jan 19;16(1):153. doi: 10.3390/v16010153 (PMC10820490; doi:10.3390/v16010153)
Supplement: Supplementary file 1 [file viruses-16-00153-s001.zip › viruses-2793809-supplementary.pdf]

Table S1. Individual characteristics and monitoring patterns of 27 wild boars captured in Lithuania in the period 2020-2022 and monitored through GPS-GSM collars.

Wild boar	Sex	Age class
8006	female	yearling
8008	male	adult
8011	male	yearling
8019	female	adult
8030	male	adult
8031	male	adult
8032	male	yearling
8036	male	adult
8037	male	adult
8039	male	juvenile
8041	male	adult
8042	male	yearling
8043	female	yearling
8045	male	yearling
211227	male	adult
216860	male	yearling
216861	male	juvenile
216862	male	juvenile
216863	male	juvenile
216865	male	yearling
216867	male	adult
8032_bis	male	yearling
8033_bis	female	yearling
8037_bis	male	yearling
8040_bis	male	yearling
8042_bis	male	adult
8043_bis	female	adult
